# *Ganoderma lucidum* polysaccharide (GLP) enhances antitumor immune response by regulating differentiation and inhibition of MDSCs via a CARD9-NF-κB-IDO pathway

**DOI:** 10.1042/BSR20201170

**Published:** 2020-06-23

**Authors:** Yongyong Wang, Xiaowu Fan, Xiaowei Wu

**Affiliations:** Department of Thoracic Surgery, Tongji Hospital, Tongji Medical College of Huazhong University of Science and Technology, Wuhan 430030, China

**Keywords:** CARD9-NF-κB-IDO pathway, Ganoderma lucidum, Lung cancer, Monocytic myeloid-derived suppressor cells (MDSCs), Polysaccharide

## Abstract

A homogeneous polysaccharide (GLP), with an average molecular weight of 4.44 × 10^4^ Da, was isolated and purified from the fruiting bodies of *Ganoderma lucidum*. In this work, we examined the antitumor activities of GLP using a mouse Lewis lung cancer (LLC) model and explored possible molecular pathways involved in its immunomodulatory mechanism on tumor–host interaction. GLP administration (25 and 100 mg/kg) significantly inhibited tumor growth, as evidenced by the decreased tumor volume and tumor weight, as well as histological features of tumor tissues with concomitant down-regulation of proliferating cell nuclear antigen (PCNA) proliferative marker. Less myeloid-derived suppressor cells (MDSCs) were accumulated in both spleen and tumor tissues from GLP-treated mice. In contrast, the percentage of CD4+ and CD8+ T cells together with the production of Th1-type cytokines (IFN-γ and IL-12) was increased in the spleen of LLC-bearing mice following GLP administration. Furthermore, GLP administration reversed the attenuated expression of CARD9, p-Syk and p-p65, and increased indoleamine 2,3-dioxygenase (IDO) protein expression in MDSCs of LLC-bearing mice. Collectively, our data demonstrated the first time that GLP induced the differentiation of MDSCs and inhibited the accumulation of MDSCs via CARD9-NF-κB-IDO pathway, thus prevented lung cancer development.

## Introduction

Lung cancer is one of the most concerned health challenges due to its high morbidity and mortality, and new cases number are rising worldwide [1]. It is estimated that among total estimated 9.6 million cancer deaths in 2018, lung cancer accounting for 2.09 million cases [2]. Despite great advances in early diagnostic detection and increased therapeutic options for lung cancer in the past two decades, many patients underwent unacceptable recurrence and progression with no virtual improvements in survival [3]. It is evident that current conventional treatments including surgery, radiation and chemotherapy have limited effectiveness and unfortunately, the prognosis remains mediocre in operated patients [4,5]. Therefore, discovering novel anticancer drugs with less side effects and new strategies for the treatment of lung cancer are so much impending.

In recent years, immunotherapy provides a new approach for lung cancer treatment [6]. Tumor-elicited immunosuppression is one of the critical mechanisms for tumor escape from immune surveillance [7]. Recent studies have suggested that myeloid-derived suppressor cells (MDSCs) accumulate in tumor-bearing hosts and are the major suppressor of the immune responses, which hampers effective immunotherapeutic approaches [8]. Screening for drugs with the ability to inhibit the deposition of MDSCs in tumor-bearing patients has received tremendous scientific attention in cancer research. As known to all, plant-based polysaccharides are wildly used in the prevention and treatment of various cancers in the world, which can enhance the body's immunity with less toxicity [9,10]. *Ganoderma lucidum* (Fr.) Karst, an edible herbal medicine and popular dietary supplement, has been used for more than 2000 years in China and other oriental countries [11]. This mushroom contains two major categories of the bioactive ingredients, namely polysaccharides and triterpenes, then phenols, steroids, amino acids, lignin, vitamins, nucleosides and so on [12]. *G. lucidum* polysaccharide (GLP), an important polysaccharide with a linear (1→3)-β-d-Glc*p* as main chain linkage in the fruiting bodies of this mushroom, aroused the interest of scientists around the world owing to its renowned pharmacological activities and antitumor effects [13]. Consistent with this notion, many studies showed that the antitumor activity of GLP was also contributed by the activation of mitogen-activated protein kinases, inhibition of angiogenesis, adhesion, migration and invasion, as well as direct cytotoxic effects, *etc*. [14–17]. Moreover, there has been a growing body of evidence to suggest that increasing the host's immune activity was believed to be one of the most important contributions for GLP as anticancer agents. The immuno-modulating effects of GLP were extensive, including promoting the function of antigen-presenting cells, mononuclear phygocyte system, humoral immunity and cellular immunity [18], which suggested that GLP can be used as an effective drug in immunotherapy. Despite many reported antitumor functions related to GLP, the molecular mechanism of antitumor activity of GLP was not well characterized due to their structure diversity. In this work, we intend to isolate GLP from the fruiting bodies of *G. lucidum*, and examine if GLP can elicit an effective antitumor immune response in a murine Lewis lung cancer (LLC) model by elimination of MDSCs and elucidate the underlying mechanism underpinning this effect.

## Materials and methods

### Materials and chemicals

The fruiting bodies of *G. lucidum* were obtained commercially from Hubei province of China. DEAE cellulose-52 and Sepharose 6 Fast Flow were purchased from the GE Healthcare Ltd. (Chalfront St. Guiles, U.K.). Antibodies against proliferating cell nuclear antigen (PCNA), caspase recruitment domain-containing protein 9 (CARD9), phosphorylated Syk (p-Syk), p-p65, indoleamine 2,3-dioxygenase (IDO) and β-actin as well as horseradish peroxidase (HRP)-conjugated secondary antibodies were obtained from Santa Cruz Biotechnology (SantaCruz, CA). Trifluoroacetic acid (TFA), l-rhamnose (Rha), l-arabinose (Ara), d-xylose (Xyl), d-mannose (Man), l-fucose (Fuc), d-galactose (Gal), d-glucose (Glc) and T-series Dextran (T-10, T-40,T-70, T-500 and T-2000) were purchased from Sigma–Aldrich Co. (St. Louis, MO, U.S.A.). GTVisin™ anti-mouse/anti-rabbit immunohistochemical analysis kit was from Gene Tech Co., Ltd. (Shanghai, China). MDSC isolation kit was from Miltenyi Biotec (Auburn, CA) IFN-γ and IL-12 assay kits were from Nangjing KeyGEN Biotech. Co., Ltd. (Nanjing, China). Murine fluorochrome-conjugated antibodies (against CD45, CD11b, Gr-1, Ly6C and Ly6G) and FITC-conjugated rat anti-mouse CD3, PerCP-CD4, PE-CD8 antibodies were from Nanjing Jiancheng Biotech. Co., Ltd. (Nanjing, China). All other chemicals and solvents were used at least of analytical grade.

### Isolation and purification of polysaccharide GLP

The dried *G. lucidum* fruiting bodies (1000 g) was refluxed with 95% ethanol in a Soxhlet apparatus for 10 h to get rid of small molecule impurities, such as pigment, lipids, monosaccharides, and so on. The refluxed residues were filtrated and extracted three times with distilled water at 90°C for 3 h each time. After extraction, the combined extracts were centrifuged (5000 ***g***, 10 min) to remove the insoluble material and concentrated to one-fifth of the original volume, followed by precipitation by the addition of ethanol (final concentration: 80%, v/v) at 4°C overnight. The precipitate collected by centrifugation (5000 ***g***, 10 min) was then dissolved in distilled water and deproteinated with Sevag reagent (1:4 n-butanol:chloroform, v/v). The final deproteinated supernatant was intensively dialyzed (cut-off Mw = 8000 Da) for 2 days against distilled water, and then precipitated with 95% ethanol to give crude polysaccharide fraction crude *G. lucidum* polysaccharide (CGLP) (86.5 ***g***, 8.65% of the starting raw material).

The crude CGLP (50 mg) was dissolved in 10 ml of distilled water (5 mg/ml), centrifuged, and then subjected to a DEAE-52 cellulose column (2.6 cm × 40 cm) sequentially eluted stepwise with 1 column volume of NaCl solutions (0, 0.25, 0.5 and 1.0 M) at a flow rate of 1.0 ml/min. The eluent fractions (5 ml/tube) were collected automatically and monitored at 490 nm for the absorbance using the phenol–sulfuric acid method [19]. Accordingly, four completely separated fractions (CGLP1, CGLP2, CGLP3 and CGLP4) were obtained from distilled water, 0.25, 0.5 and 1.0 M NaCl, respectively. The resulting major water-eluted fraction CGLP1 was further loaded onto a column (2.6 cm × 100 cm) of Sepharose 6 Fast Flow eluted with 0.15 M NaCl at a flow rate of 1 ml/min, yielding a purified polysaccharide fraction (GLP, 7.98 g, 0.8% of the starting raw material), which was used for the subsequent assays.

### Chemical characterization, molecular weight and monosaccharide composition of polysaccharide GLP

The total sugar and uronic acid contents were determined by the phenol–H_2_SO_4_ method using galactose as the standard [19] and m-hydroxydiphenyl colorimetric method using galacturonic acid as the standard [20], respectively. Protein content was measured by Bradford methods using bovine serum albumin (BSA) as the standard [21].

The homogeneity and the average molecular weight of the polysaccharide were determined using high performance size exclusion chromatography (HPSEC) on a Shimadzu LC-2010A HPLC system (Shimadzu Corp., Japan) coupled with a TSKGel G3000SWxl column (7.5 mm × 300 mm, 5 μm, Tosoh Bioscience LLC, PA, U.S.A.) and a refractive index detector (RID-10A, Shimadzu). The polysaccharide samples were totally dissolved in distilled water (10 mg/ml) and passed through a 0.22 μm filter. A 20 μl aliquot was injected for each run with 0.1 M MNa_2_SO_4_ as the mobile phase at a flow rate of 0.8 ml/min. The average molecular weight was estimated by referring to the calibration curve, which was made using different molecular weight of T-series Dextran standards (DextranT-10, T-40, T-70, T-500 and T-2000).

The monosaccharide composition of the polysaccharide was performed by gas chromatography (GC) analysis, as described by Sun et al. [22], with minor modifications. Briefly, a unit of GLP sample (10 mg) was hydrolyzed with 2 ml of 2 M TFA at 120°C for 2 h in a sealed test tube. After the hydrolysis, the reaction mixture was evaporated with methyl alcohol (MeOH) under reduced pressure to get rid of TFA, reduced by NaBH_4_, and then subjected to alditol acetates conversion with acetic anhydride. The monosaccharide composition of the polysaccharide was determined on a Agilent 6890 N GC instrument (Santa Clara, CA, U.S.A.) fitted with flame ionization detector (FID) and a HP-5 column (30 m × 0.32 mm × 0.25 μm) according to retention time of the GC–MS alditol acetates of standard monosaccharides (Rha, Ara, Xyl, Ma, Gal, Glc, GalA and GlcA) with inositol as the internal standard.

### Cell line

Mouse LLC cells were obtained from Cell Bank of Type Culture Collection of Chinese Academy of Sciences (Shanghai, China) and maintained in RPMI 1640 medium supplemented with 10% FCS, 100 U/ml penicillin and 100 μg/ml streptomycin at 37°C under a humidified atmosphere containing 5% CO_2_ (v/v). Lewis cells yielding 80–90% confluence were prepared to suspension for animal experiment.

### Animals and Lewis lung cancer model

Male C57BL/6 mice aged 6–8 weeks (18–22 g) were purchased from the Experimental Animal Center at Tongji Medical College of Huazhong University of Science and Technology, Wuhan, China and housed for 1 week under a 12 h light/12 h dark cycle in a pathogen-free environment with a humidity of 60 ± 10%. They had free access to food and water *ad libitum*. All animal experiment protocols were performed and approved by the Institutional Animal Care and Use Committee of Huazhong University of Science and Technology.

A single LLC cell suspension (5 × 10^5^) in 0.1 ml of PBS was injected subcutaneously into the right flank of each mouse. When the tumor volume grown to approximately 50 mm^3^, 40 tumor-bearing mice were randomly assigned to four groups (10 per group) and subsequently administered for 14 consecutive days. Two groups of C57BL/6 mice were intragastrically administrated with 25 and 100 mg/kg of GLP every day using an intragastric gavage needle and the mice in the negative control group received an equal volume of saline solution (0.9%) at the same time. Cisplatin (1 mg/kg body weight), serving as positive control, was given every 3 days via intraperitoneal injection. Another 10 normal mice were used as normal control and treated as negative control. Tumor volumes of mice were measured every other day and calculated using the following formula: $\;\text{Tumor volume }=\;0.5\;\times \text{ L}\;\times \;{\text{W}}^{2}$, where ‘*L*’ is larger tumor diameters and ‘*W*’ is smaller tumor diameters. After being killed by cervical dislocation and weighted, the tumor and the major immune organs including thymus and spleen were excised and their weights were determined immediately. The tumor growth inhibition rate was calculated using the following formula: Tumor inhibitory ratio (%) = [(A−B)/A] × 100%, where *A* and *B* represent the average tumor weights of the negative (untreated) and treated groups, respectively [23].

### Histological examination

Tumor tissue samples dissected from mice were fixed in 4% paraformaldehyde (pH 7.5) for 4 h, embedded in paraffin, cut into sections with thickness of 4 μm and stained with hematoxylin and eosin (H&E) stain. The staining section slides were photographed under a IX73 microscopy (Olympus, Tokyo, Japan).

### Immunohistochemistry

Paraffin-embedded tumor sections (4 μm) were deparaffinized, rehydrated and then treated with of Proteinase K (20 mg/ml), followed by the addition of hydrogen peroxide (3%) to block the endogenous peroxidase activity. Thereafter, the section was incubated with anti-PCNA antibody at a 1:400 dilution for 1 h and detected with GTVisin™ anti-mouse/anti-rabbit immunohistochemical analysis kit. Finally the images were visualized under a IX73 microscopy (Olympus, Tokyo, Japan).

### Preparation of single cell suspension from spleen and tumors

Splenocytes eluent was obtained from different groups by PBS washing, and then passed through a 200-mesh sieve to yield single cell suspension. Tumors acquired from different groups were minced into small (1–2 mm^3^) pieces and digested with enzyme. The resulting mixture was incubated at 37°C for 2 h on a rotating platform and then the supernatant was filtered through a 200-mesh sieve and washed twice with ice-cold PBS. Remaining red blood cells were eliminated by suspending on ammonium chloride solution.

### Isolation of MDSCs

Murine MDSCs were isolated from the spleens of tumor-free control or LLC tumor bearing mice using a mouse MDSC isolation kit in accordance with the manufacturer's protocol.

### Flow cytometry

Single cell suspension of spleen or tumors was labeled with relevant murine fluorochrome-conjugated CD45, CD11b, Gr-1, Ly6C and Ly6G antibodies for detecting MDSCs. Single cell suspension of spleen was incubated with FITC-conjugated rat anti-mouse CD3, PerCP-CD4, PE-CD8 for measuring CD4+, CD8+ T cells. Staining was performed at 4°C for 1 h and then stained cells were washed, centrifuged (380 ***g*** for 5 min) and resuspended with 200 μl PBS for immediate flow cytometric analysis. The percentage of positively stained cells was performed on the FACSCalibur flow cytometer (Becton Dickinson, Franklin Lakes, NJ) and analyzed with FlowJo software (Treestar, Inc., San Carlos, CA) over 10000 events.

### Detection of ROS levels, arginase activity and NO production

ROS production in tumor tissues was analyzed by flow cytometry using the ROS-sensitive fluorescent probe 2′,7′-dichlorofluorescin diacetate (DCFH-DA) as previously described [24]. Briefly, cell suspension from tumors was pre-loaded with 5 μM DCFH-DA for 30 min at 4°C and then washed before re-suspension in ice-cold PBS. Finally the DCF fluorescence intensity formed by reaction of DCF-DA with intracellular ROS was measured by flow cytometry (excitation at 488 nm; emission at 525 nm).

Arginase activity and the amount of NO were monitored using a quantitative QuantiChrom Arginase Assay kit and the colorimetric nitrite assay kit (Griess Reaction) as per respective manufacturer's protocols.

### Enzyme-linked immunosorbent assay

The IFN-γ and IL-12 levels in the supernatants from cultured lymphocytes were determined by respective ELISA kits.

### Western blot

Western blot assay was performed as previously reported [25] with slight modifications. After incubation, the MDSCs isolated from difference group were washed three times with ice-cold PBS and suspended in RIPA lysis buffer for total cell protein extraction. Cell lysates were centrifuged at 12000 rpm for 10 min at 4°C and protein concentrations in supernatants were determined using a BCA protein assay kit. Equal quantities of protein (20 μg) were separated on a 12% sodium dodecyl sulfate/polyacrylamide gel electrophoresis gel (SDS/PAGE) gel, and then transferred onto polyvinylidene fluoride (PVDF) membranes. After that, membranes were blocked using 5% (w/v) non-fat milk in TBST for 1 h at room temperature with a gently shaking and probed with primary antibodies (CARD9, p-Syk, p-p65, IDO and β-actin) at 1:3000 dilutions overnight at 4 C, followed by incubation for 1 h with the secondary antibodies conjugated with HRP (1:3000 dilution) at the room temperature. After four additional washes with TBST, the blots were visualized using enhanced chemiluminescent (ECL) reagents. In order to quantify changes of protein expression in each lane, the target protein was normalized against β-actin, which was selected as an internal reference.

### Statistical analysis

Data were expressed as mean±S.D. and analyzed using two-way analysis of variance (ANOVA) and Student's *t*-test with Prism 5 (GraphPad Software, Inc., San Diego, CA). A *P* value of less than 0.05 was considered statistically significant.

## Results

### Isolation and characterization of the polysaccharide GLP

**Figure 1:**
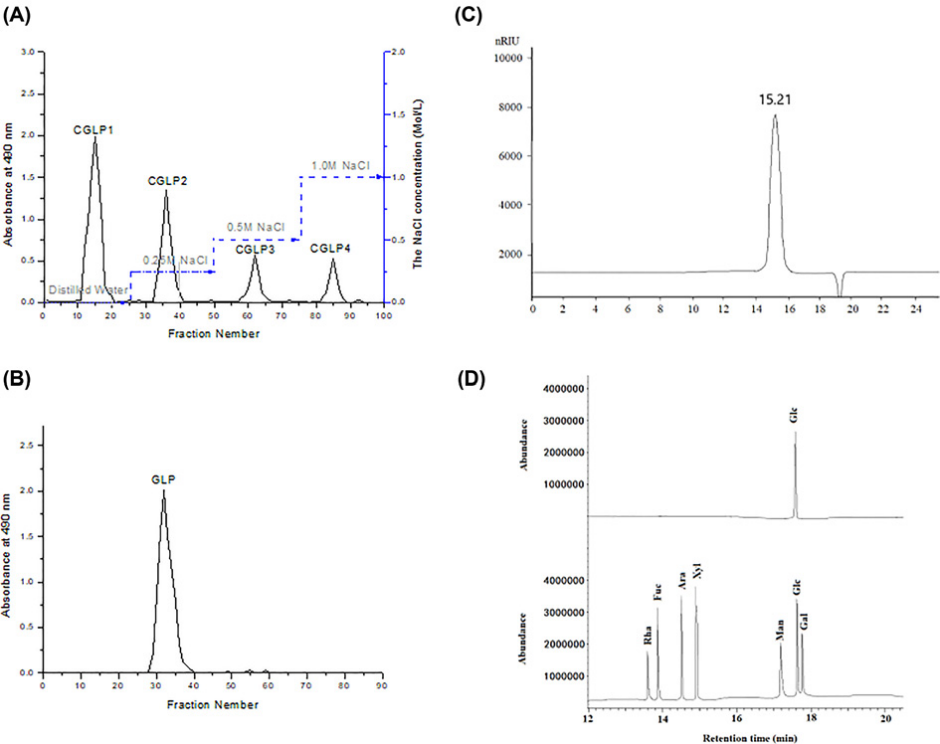
Isolation and characterization of the polysaccharide GLP (**A**) Elution profile of CGLP by anion exchange chromatography on a column of DEAE cellulose-52. (**B**) Elution profile of CGLP1 by size exclusive chromatography on a column of Sepharose 6 Fast Flow. (**C**) Elution profile of GLP in HPSEC. (**D**) GC–MS of analysis of the monosaccharide composition of GLP and standard monosaccharides. Rha: l-rhamnose, Ara: l-arabinose, Xyl: d-xylose, Man: d-mannose, Fuc: l-fucose, Gal: d-galactose and Glc: d-glucose.

Crude polysaccharide fraction CGLP was isolated from the fruiting bodies of *G. lucidum* with a yield of 8.65% (on a dry weight basis) by 95% ethanol reflux extraction, hot water extraction, ethanol precipitation, Sevag reagent extraction and dialysis. CGLP was first separated through anion-exchange chromatography of DEAE-52 column and four fractions (CGLP1, CGLP2, CGLP3 and CGLP4) were collected as the eluent under the elution of distilled water, 0.25, 0.5 and 1.0 M NaCl, respectively (Figure 1A). CGLP1 with the high yield eluted by distilled water was further purified by a Sepharose 6 Fast Flow column to yield a single elution peak (Figure 1B), named as GLP, which was used for further study. The yield of GLP was 0.8% of the starting raw material. The content of total carbohydrate was 95.5% and no uronic acid was observed. The negative response to the Brad-ford assay and the absence of absorption at 280 nm of UV spectrum indicated that no protein in GLP. HPSEC elution profile of GLP (Figure 1C) displayed a single and symmetrically peak, indicating GLP was a homogeneous polysaccharide with an average molecular weight of 4.44 × 10^4^ Da (log Mw = 9.3652–0.3102*t*. *t* = 15.21 min, *R*^2^ = 0.9992). Monosaccharide compositional results (Figure 1D) indicated that Glc was the major monosaccharide in GLP.

### GLP inhibited tumor growth of mice

**Figure 2:**
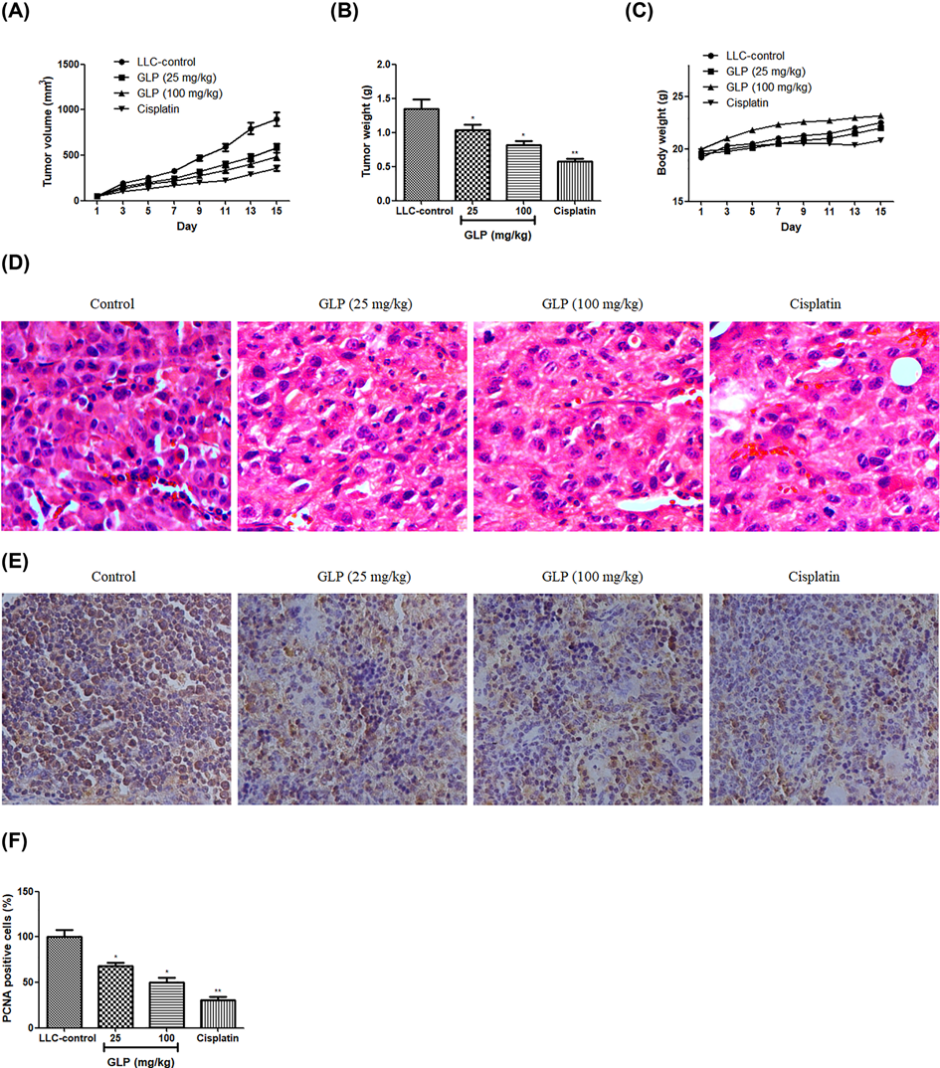
GLP inhibited tumor growth of mice (**A**) Effect of GLP on the tumor volumes of LLC inoculated onto the flank of C57BL/6 mice. (**B**) Effect of GLP on the tumor weight of LLC inoculated onto the flank of C57BL/6 mice. (**C**) Effect of GLP on the body weight of C57BL/6 mice. (**D**) Paraffin sections of tumor tissues from mice were analyzed by H&E staining (200× magnifications). (**E**) Paraffin sections of tumor tissues from mice were analyzed by immunohistochemistry of PCNA (100× magnifications). (**F**) Quantitative result of immunohistochemistry of PCNA. Results are expressed as mean + S.D. of *n* = 10 representative of three independent experiments performed. *P <0.05, **P <0.01 vs. LLC-control.

To examine whether GLP exacerbates or suppresses tumor growth *in vivo*, an animal model implanted with LLC cells in C57BL/6 mice was established. As depicted in Figure 2A,B, administration of GLP (25 and 100 mg/kg) significantly reduced both the tumor size and tumor weight as compare with the negative control (*P*<0.05). Of note, the tumor volume dropped from 899 mm^3^ (control group) to 580 mm^3^ (25 mg/kg-treated group), 487 mm^3^ (100 mg/kg-treated group) and 354 mm^3^ (cisplatin-treated group) at the end of the experiment. Also, the tumor weight dropped from 1.35 g (control group) to 1.04 g (25 mg/kg-treated group), 0.82 g (100 mg/kg-treated group) and 0.58 g (cisplatin-treated group). At the end of points, it is also observed that a slight body weight loss in cisplatin treated mice, whereas, no significant change was recorded in GLP-treated mice (Figure 2C). To further verify the inhibition effect of GLP on the tumor growth, histological examination and the proliferation in tumor tissues were examined by H&E staining and PCNA immunohistochemistry analysis, respectively. A typical abundant mitosis and compact tumor cells were shown in H&E stained-section from control group while the tumor cells were sparse and separated in the tumor sections of mice received with treatment of GLP (25 and 100 mg/kg) or cisplatin (Figure 2D). Next, we detected the proliferative marker PCNA for tumor growth using immunohistochemistry analysis. The result in Figure 2E,F showed that GLP (25 and 100 mg/kg) or cisplatin treatment significantly decreased the levels of PCNA compared with negative control (*P*<0.05 or *P*<0.01). These data suggest that GLP can obviously delay tumor progression and has fewer side effects.

### GLP reduced the accumulation of MDSCs in spleen and tumor tissue of mice

**Figure 3:**
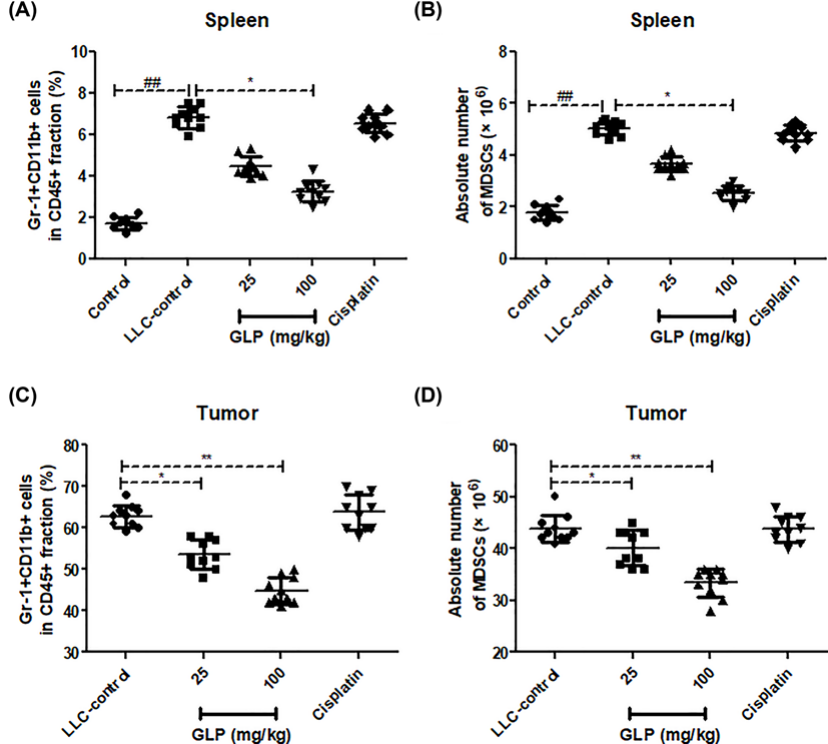
GLP reduced the accumulation of MDSCs in spleen and tumor tissue of mice (**A**) Effect of GLP on the percentage of MDSCs in the spleen of mice. (**B**) Effect of GLP on the absolute number of MDSCs in the spleen of mice. (**C**) Effect of GLP on the percentage of MDSCs in the tumor tissues of mice. (**D**) Effect of GLP on the absolute number of MDSCs in the tumor tissues of mice. Results are expressed as mean + S.D. of *n* = 10 representative of three independent experiments performed. *P <0.05, **P <0.01 vs. LLC-control. ^##^*P* <0.01 vs. control.

We next examined the status of MDSCs accumulation in both spleen and tumor tissues from different group. As seen from (Figure 3), it was evident that MDSC deposition was more in spleen of LLC-bearing mice control compared with tumor free mice (*P*<0.01), whereas GLP (25 and 100 mg/kg) treatment significantly reduced the percentage of this proportion in both spleen and tumor tissues compared with that of LLC-controls (*P*<0.05 or *P*<0.01), which suggested GLP can obviously inhibit tumor progression by suppressing MDSCs accumulation.

### GLP increased the percentage of CD4+, CD8+ T cells and their IFN-γ and IL-12 production in spleen of mice

**Figure 4:**
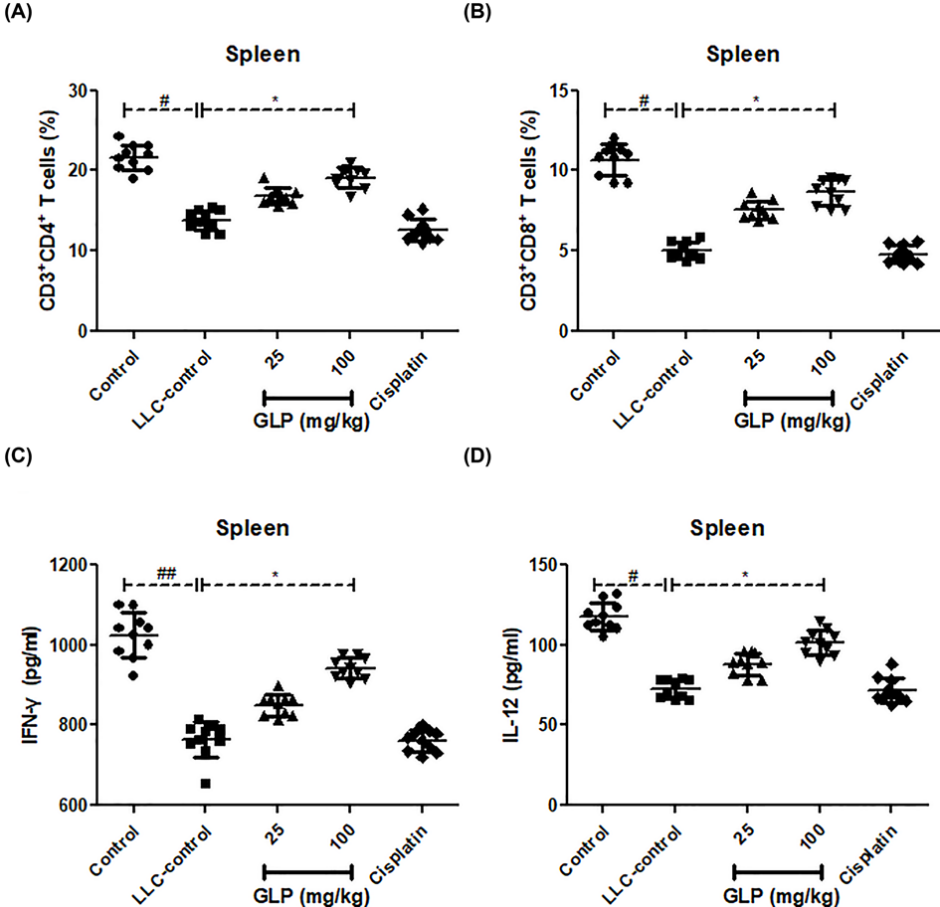
GLP increased the percentage of CD4+, CD8+ T cells and their IFN-γ and IL-12 production in spleen of mice (**A**) Effect of GLP on the percentage of CD4^+^ T cells in the spleen of mice. (**B**) Effect of GLP on the percentage of CD8^+^ T cells in the spleen of mice. (**C**) Effect of GLP on the level of IFN-γ in the spleen of mice. (**D**) Effect of GLP on the level of IL-12 in the spleen of mice. Results are expressed as mean + S.D. of *n* = 10 representative of three independent experiments performed. *P <0.05 vs. LLC-control. ^#^*P* <0.05, ^##^*P* <0.01 vs. control.

To examine whether antitumor effect of GLP was associated with its immunological enhancement in tumor-bearing mice, we analyzed the proportion of CD4^+^ and CD8^+^ T cells as well as the level of IFN-γ and IL-12 in spleens from mice. As depicted in Figure 4A,B, the number of CD4+ and CD8+T cells in the spleen of LLC-bearing mice was much less than that in normal mice (*P*<0.05). This reduction was definitely reversed following GLP administration at doses of 25 and 100 mg/kg as compared with LLC-control (*P*<0.05). Also, the reduced level of IFN-γ and IL-12 in spleen of LLC-bearing mice was significantly enhanced by GLP treatment when compared with that in LLC-control (Figure 4C,D, *P*<0.05). There was not any change for these parameters between LLC-bearing control mice and cisplatin-treated mice (*P*>0.05). This data indicated that GLP could increase the proportions of both CD4^+^ and CD8^+^ T lymphocytes, that secret cytokines IFN-γ and IL-12 to enhance immune-enhancing activity.

### GLP decreased arginase activity and NO production, but increased IL-12 production in tumor tissue of mice

**Figure 5:**
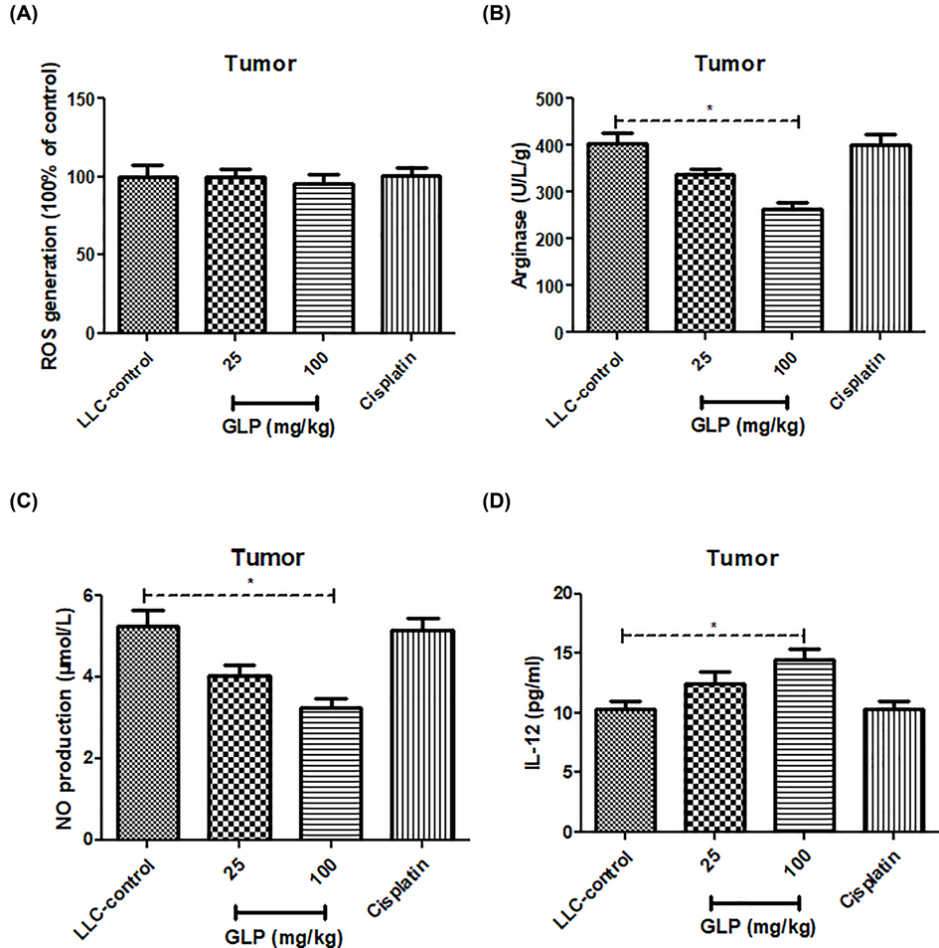
GLP decreased ROS levels, arginase activity, NO production and IL-12 production in tumor tissue of mice (**A**) Effect of GLP on the production of ROS in the tumor tissues of mice. (**B**) Effect of GLP on the arginase activity in the tumor tissues of mice. (**C**) Effect of GLP on the production of NO in the tumor tissues of mice. (**D**) Effect of GLP on the production of IL-12 in the tumor tissues of mice. Results are expressed as mean + S.D. of *n* = 10 representative of three independent experiments performed. *P <0.05 vs. LLC-control.

Due to the inhibitory ability of GLP on the population of MDSCs, several MDSCs indicators such as the levels of ROS, NO and IL-12, as well as arginase activity were determined in tumor specimens. As illustrated in Figure 5A, no difference of ROS levels was observed between LLC-bearing mice treated with or without GLP (*P*>0.05). The arginase activity and NO level in tumor tissues were virtually decreased after administration of GLP (25 and 100 mg/kg) when compared with those in LLC-bearing mice (Figure 5B,C, *P*<0.05), whereas the level of IL-12 was higher than LLC-bearing mice (Figure 5D, *P*<0.05). This result further supported the fact that the antitumor effect of GLP was achieved by repressing MDSCs population.

### GLP increased the protein expression of CARD9, p-Syk and p-NF-κB p65, but decreased the protein expression of IDO in MDSCs of mice

**Figure 6:**
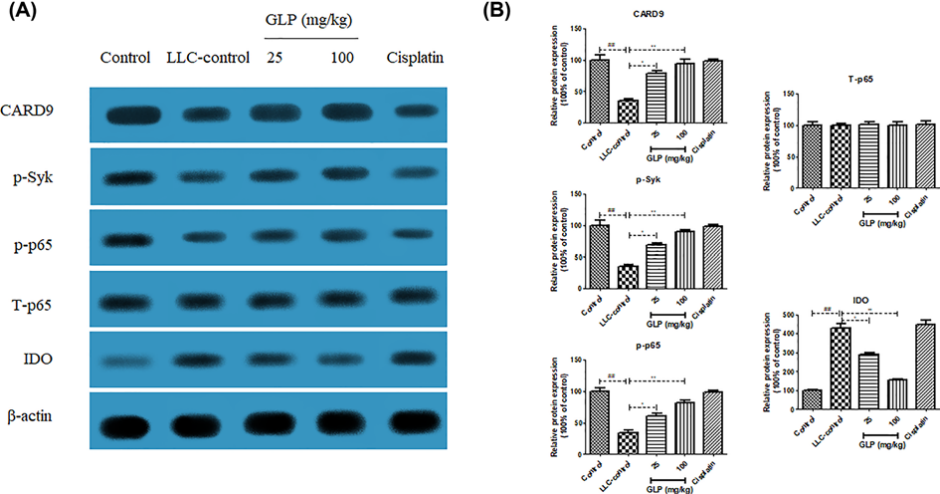
GLP increased the protein expression of CARD9, p-Syk and p-NF-κB p65, but decreased the protein expression of IDO in MDSCs of mice (**A**) Effect of GLP on the protein expression of CARD9, p-Syk, p-p65 and IDO in MDSCs of mice. (**B**) Quantitative result of the protein expression of CARD9, p-Syk, p-p65 and IDO in MDSCs of mice. Results are expressed as mean + S.D. of *n* = 10 representative of three independent experiments performed. *P <0.05, **P <0.01 vs. LLC-control. ^##^*P* <0.01 vs. control.

To investigate possible molecular mechanisms responsible for positive effect of GLP on MDSC differentiation, western blot analysis was used to determine the protein expression of CARD9, p-Syk, p-p65 and IDO in MDSCs from different treatment. The results in Figure 6A,B revealed that the expression of CARD9, p-Syk and p-p65 protein in MDSCs from tumor-free mice was higher than those from LLC-bearing mice (*P*<0.01). In contrast, IDO protein expression in MDSCs from tumor-free mice was lower than that from LLC-bearing mice (*P*<0.01). This tendency was turned to the opposite in GLP-treated groups (25 and 100 mg/kg) and their expression was significantly different from those in MDSCs from LLC-bearing mice (*P*<0.05 or *P*<0.01). In addition, total p65 (T-p65) kept unchanged in all groups. This data indicated that GLP worked on CARD9-NF-κB-IDO pathway to differentiate MDSCs.

## Discussion and conclusion

The tumor immune microenvironment is a complex milieu in which the interplay between tumor cells and the host immune response may result in a malignancy progression [26]. Recent studies have increasingly emphasized its role in cancer research within the past few years [27]. High cancer occurrences are inherently from a state of local and systemic immunosuppression associated with cancer progression in the body [28]. Thus, any drug that could alleviate this tumor-elicited immunosuppression by blocking ‘immune checkpoint’ activation would contribute to the outcome of cancer immunotherapy.

In present work, a homogeneous polysaccharide GLP, with an average molecular weight of 4.44 ×10^4^ Da, was isolated and purified from the fruiting bodies of *G. lucidum*. GC analysis showed that it was predominantly composed of Glc, which was consistent with the findings by He et al. [13] and Liu et al. [29]. To examine the antitumor activity of GLP in vivo, C57BL/6 mice were implanted with 5 × 10^5^ LLC cells into the right flank of each mouse. After the tumor grown into 50 mm^3^, the mice were treated with PBS or GLP (25 and 100 mg/kg) for another 2 weeks. GLP Administration at the doses of 25 and 100 mg/kg showed significant effect on the tumor size and tumor weight, which was comparable to the cisplatin positive control. At the same time, no body weight change was observed in all groups, except that a not significant less in cisplatin-treated group. These results indicated that GLP treatment with an optimum dosage had an antitumor effect without toxicity in mice. Next, the antitumor efficacy of GLP was confirmed by examining tumor tissues with H&E staining and immunohistochemistry analysis using PCNA antibodies. H&E staining results of resected tumor specimens from control mice showed typical pathological characteristics of malignancy evidenced by compact tumor cells, which indicated proliferating cells, whereas the structure of the tumor tissue was more seriously damaged with a limited number of mitotic-positive cells in tumor sections from GLP or cisplatin-treated group. Immunohistochemical PCNA analysis showed that GLP (25 and 100 mg/kg) treated mice had significantly lower cell proliferative index compared with the vehicle-treated animals, which was comparable to cisplatin treatment. These data further suggested that GLP can inhibit tumor growth through inhibition of cell proliferation.

MDSCs are the major population of cells with immunosuppressive activity and accumulate in tumor-bearing hosts, which play a pivotal role in tumor-induced immunosuppression [30,31]. Indeed, accumulating evidence has suggested that MDSCs quickly differentiate into mature DCs, macrophages or granulocytes in healthy individuals. In contrast, MDSCs are known to expand rapidly in pathological cases due to a partially block in the differentiation of MDSCs into myeloid cells [32]. To further investigate the underlying mechanisms of GLP in suppression of tumor growth in this lung cancer model, we determined the number of immunosuppressive MDSCs in both spleen and tumor tissues using CD45, CD11b and Gr-1 antibodies on a flow cytometry. The result showed that CD11b+Gr1+ MDSCs were more abundant in the spleen of LLC-bearing mice than in tumor-free mice. However, GLP treatment drastically decreased the frequencies of MDSCs in both spleen and tumor tissue.

In consistent with this observing, it is likely proposed that the mitigation of MDSCs in GLP-treated groups was related with the differentiation of MDSCs. It is well-acknowledged that IL-12 is a potent inducer of IFN-γ production, which stimulates T helper type 1 (Th1) effector cells (CD4+ T cells) [33]. With the help of activated CD4+ T cells, CD8+ T cells can be induced to be capable of killing the tumor cells [34,35]. To test our hypothesis, the percentage of CD4+ and CD8+ T, and the production of Th1-type cytokines (IFN-γ and IL-12) were measured in spleen of tumor bearing mice. Strikingly, the percentages of CD4+ and CD8+ T cells together with the level of IFN-γ and IL-12, which were significant reduced in LLC-bearing mice, were also restored after GLP treatment for 14 days. Probably, this was due to the generation of mature myeloid cells by MDSCs differentiation.

Substantial experimental evidence indicates that cross-talk between cancer cells and host cells in a complex microenvironment may result in an invasive behavior of tumor cells [36]. CARD9 is mainly expressed in myeloid cells and works as an important adaptor protein of innate immunity [7], which was also regarded as a crucial downstream adaptor linking Syk-coupled receptors to the NF-κB pathway [37]. Aberrant activation of CARD9 usually causes a series of inflammatory diseases or cancers. Qu et al. demonstrated the critical role of CARD9 in the development of LC for the first time [38] and draw a conclusion that CARD9 prevented lung cancer development by suppressing the accumulation of immunosuppressive cells MDSCs and its downstream product IDO production. Moreover, correlations between CARD9 expressions and MDSCs relative genes were further confirmed in tumor tissues from lung cancer patients. Strikingly, Tian et al. proved that the differentiation of MDSCs was strongly inhibited after the deficiency of canonical NF-κB pathway [32]. These findings lead us to hypothesize that the differentiation of MDSCs is mediated by the CARD9-NF-κB-IDO pathway. To investigate if GLP induces differentiation of MDSCs via CARD9-NF-κB-IDO pathway, we examined the expression of CARD9, p-Syk, p-p65 and IDO in MDSCs from different group. The decreased protein expression of CARD9, p-Syk and p65, as well as the increased IDO protein expression in MDSCs from LLC-bearing mice were totally reversed by GLP (25 and 100 mg/kg) treatment. This result suggested that the regulatory effect of GLP on differentiation of MDSCs was achieved via a CARD9-NF-κB-IDO pathway.

In summary, our work provided novel *in vivo* evidence that the antitumour activity of GLP might be due to the inhibition and differentiation of immunosuppressive cells MDSCs via a CARD9-NF-κB-IDO pathway, which gave useful information to support the possible use of GP in the clinic for immunotherapy of lung cancer.

## Abbreviations

Ara: l-arabinose
CARD9: caspase recruitment domain-containing protein 9
CGLP: crude *Ganoderma lucidum* polysaccharide
Gal: d-galactose
GC: gas chromatography
Glc: d-glucose
GLP: *Ganoderma lucidum* polysaccharide
H&E: hematoxylin and eosin
HPSEC: high performance size exclusion chromatography
HRP: horseradish peroxidase
IDO: indoleamine 2,3-dioxygenase
LLC: Lewis lung cancer
Man: d-mannose
MDSC: myeloid-derived suppressor cell
p-Syk: phosphorylated Syk
PCNA: proliferating cell nuclear antigen
Rha: l-rhamnose
TFA: trifluoroacetic acid
Th1: T helper type 1
Xyl: d-xylose
